# Stable Characteristics Optimization of Anti-Symmetric Cylindrical Shell with Laminated Carbon Fiber Composite

**DOI:** 10.3390/ma15030933

**Published:** 2022-01-26

**Authors:** Min Sun, Huping Zhou, Chongjie Liao, Zheng Zhang, Guang Zhang, Shaofei Jiang, Feng Zhang

**Affiliations:** 1 College of Mechanical Engineering, Zhejiang University of Technology, Hangzhou 310014, China; sunmin@zjut.edu.cn (M.S.); 15397077948@139.com (H.Z.); chong96524@foxmail.com (C.L.); guangzhang@zjut.edu.cn (G.Z.); Jsf75@zjut.edu.cn (S.J.); 2 Key Laboratory of Special Purpose Equipment and Advanced Processing Technology, Ministry of Education and Zhejiang Province, Zhejiang University of Technology, Hangzhou 310014, China; 3 School of Mechanics, Civil Engineering and Architecture, Northwestern Polytechnical University, Xi’an 710129, China; nwpuwindy@nwpu.edu.cn

**Keywords:** multi-objective optimization, NSGA-II, surrogate model, anti-symmetric cylindrical shell, laminated carbon fiber composite

## Abstract

This paper proposes a multi-objective optimization model for anti-symmetric cylindrical shell in the bionic gripper structure. Here, the response surface method is used to establish multiple surrogate models of the anti-symmetric cylindrical shell, and the non-dominated sorting genetic algorithm-II (NSGA-II) is used to optimize the design space of the anti-symmetric cylindrical shell; the design points of the anti-symmetric cylindrical shell are verified by experimental methods. The optimization goals are that the first steady state transition load (the transition process of the bionic gripper structure from the open state to the closed state) of the anti-symmetric cylindrical shell is minimized, and the second steady state transition load (the transition process of the bionic gripper structure from the closed state to the open state) is the largest. At the same time, in order to prevent stable instability caused by stress concentration in the second steady state of the anti-symmetric cylindrical shell, the maximum principal plane stress is given as the constraint condition. The validity of the optimization results is verified by finite element and experimental methods. Due to the stable transition load of the anti-symmetric cylindrical shell being significantly larger than that of the orthogonal laminated plate, therefore, the anti-symmetric cylindrical shell has potential application prospects in the application of deformable structures and bionic structures that require composite functions such as having light weight, high strength, and large clamping force. The novelty of this paper lies in the multi-objective optimization of the application of the antisymmetric bistable cylindrical shell in the bionic gripper structure.

## 1. Introduction

Intelligent composite structures, such as bistable composite structures [1], deployable composite structures [2] and composite grid structures [3], have good load-bearing capacity, deformability, and light weight [4], which are used in many advanced engineering fields. In particular, the bistable structure can maintain two stable configurations without requiring a continuous power source, which has attracted more and more attention from researchers all over the world, and it has been proposed for the development of deformable structures in the aerospace field [5] as having great potential. The bistable composite structure [6,7,8,9] is divided into anti-symmetric and orthogonal bistable structures according to different laying methods of materials.

Orthogonal bistable structure is widely used in deformable wings, gripper structures, and energy harvesting devices due to its advantages of light weight, excellent mechanical properties, and high space utilization. Many scholars have also conducted research on the optimal design of orthogonal bistable structures. Hufenbach [10] adopted a combination of genetic algorithm and nonlinear calculation method to adjust the out-of-plane deformation of the orthogonal bistable structure, which is caused by uneven residual stress caused by thermal effects, humidity effects, and chemical shrinkage. Betts [11] perfected the theoretical model of orthogonal bistable structure, defined the bending stiffness of the orthogonal bistable structure in two directions, and optimized the rigidity of the orthogonal bistable structure by using the quadratic sequence method. The deflection between the two steady states is used as a constraint condition, and the extreme value of the bending stiffness in the two directions is taken. After that, Betts et al. optimized the design of the device based on MFC and orthogonal bistable structure through the above optimization process, so as to realize driving with low voltage. Panesar [12] used ant colony algorithm and finite element method to maximize the out-of-plane displacement as the design goal, and designed the deformable wing based on the orthogonal bistable structure by changing the ply angle of different areas. Kuder [13] proposed a parallel design and optimization framework based on genetic algorithms to design a multi-mode deformable wing, and realized different deformation modes of the deformable wing through a bistable laminate structure. Haldar [14] proposed an optimization design framework based on a global pattern search algorithm for wind turbine rotor blades embedded with bistable laminates, and simulated, analyzed, and verified the optimization results through the finite element method.

Compared with orthogonal laminated plates, there are fewer studies on anti-symmetric cylindrical shells. Zhang [15] carried out a systematic study on anti-symmetric cylindrical shells, considering the influence of various factors of laminated plates on the bistable characteristics. Then, they [16,17] combined the anti-symmetric carbon fiber reinforced cylindrical shell with a non-contact electromagnetic drive method, and proposed a bistable artificial leaf imitating Venus flytrap gripper, and used finite element methods and experimental methods to study. Later, a new type of anti-icing/de-icing system composed of a bistable laminated composite structure of super-hydrophobic surface and soft electric heating patch was proposed. Belbachir [18] obtained an anti-symmetric cylindrical shell in a steel semi-cylindrical mold through high temperature holding pressure and solidification, and then performed a bending analysis. Santo [19] combined the mechanical properties of composite materials with the functional behavior of shape memory polymers, and simulated the mechanical behavior of a retractable solar sail with an SMC frame through finite element modeling. Viswanathan [20] studied the free vibration of the anti-symmetric cylindrical shell wall under the first-order shear deformation theory by the spline method, and analyzed the parameters of length, the number of circumferential nodes, the angle of the laminate, the number of laminates, the laminated material, and the boundary conditions of the anti-symmetric cylindrical shell. Baharali [21] studied the effects of carbon nanotubes (CNTs) and fiber carbon on the vibration frequency of anti-symmetric cylindrical shells, and evaluated the effects of carbon nanotube weight percentage and geometric parameters. Narwariya [22,23] conducted numerical studies on the free vibration and harmonic analysis of anti-symmetric laminates, and obtained the frequency-amplitude relationship of anti-symmetric anisotropic plates. Garg [24] studied the static response of anti-symmetric laminated composites under high temperature and humidity conditions, considering various parameters such as the influence of heat and humidity coefficients, material anisotropy, boundary and load conditions, and span-to-thickness ratio. In the optimization of anti-symmetric cylindrical shells, Barroso [25] proposed a new hybrid particle swarm optimization (PSO) to optimize the structure of composite laminated structures, with the goal of maximizing strength and minimizing weight. Coelho [26] used a multi-scale topology optimization model to optimize the design of the orthogonally laid laminated structure and its materials, to obtain the best composite microstructure at the micro design level and the best fiber orientation at the macro level. Reddy [27] adopted the newly developed Enhanced Bat Algorithm (EBA) to perform the minimum weight optimization of laminated composites, and used an unconventional stacking sequence to increase the damage tolerance of the laminate. Aydin [28] used an effective global optimization method to design dimensionally stable laminated composite materials to obtain high stiffness and low thermal expansion coefficient and moisture expansion coefficient. The proposed optimization algorithm has also been experimentally verified. At present, there are relatively few optimization studies on the application of bistable cylindrical shells in bionic gripper structure, and the geometric characteristics and mechanical properties of cylindrical shells have a great influence on the gripping performance of bionic gripper structure.

In this paper, a multi-objective optimization method of the anti-symmetric cylindrical shell will be proposed for application in the bionic gripper structure. The two-point loading method is used to simulate the snap behavior of anti-symmetric cylindrical shells and obtain the driving force. A finite element model, which demonstrates the geometric shape and snap process of the anti-symmetric cylindrical shell, is shown in Section 2. Section 3 shows the multi-objective optimization model based on NSGA-II algorithm. The optimization results will be verified in Section 4 by experimental method. Finally, the conclusion of the paper can be found in Section 5.

## 2. Anti-Symmetric Cylindrical Shell

### 2.1. Finite Element Model

**Figure 1:**
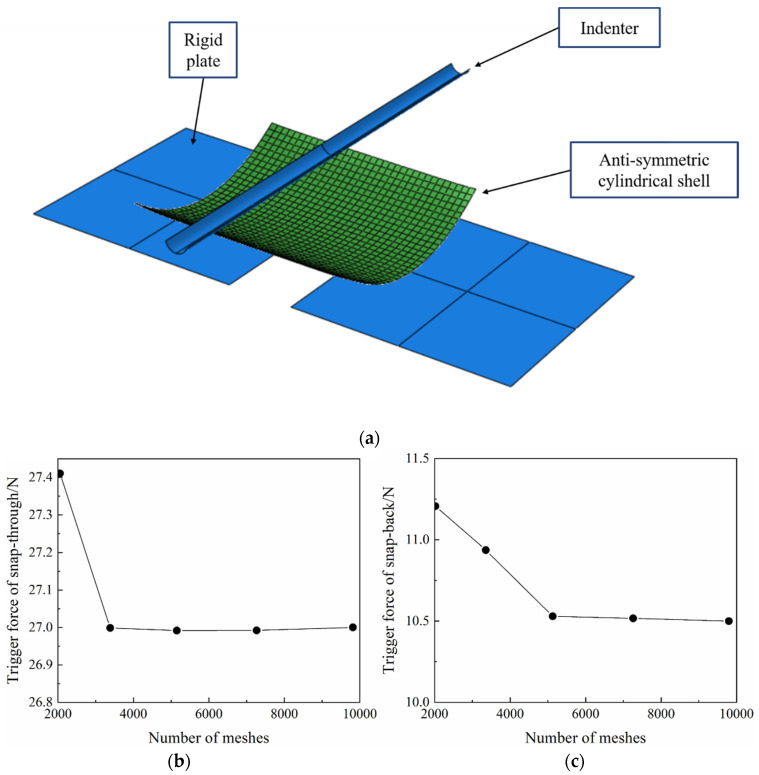
Anti-symmetric shells finite element model and grid independence analysis: (**a**) The finite element model, (**b**) first steady state, (**c**) second steady state.

Figure 1a shows the finite element model of the anti-symmetric cylindrical shell, which achieves stable transformation by pressing down the indenter. There are snap-through and snap-back in the anti-symmetric cylindrical shell during the modeling process [29,30,31,32]. During the snap-through process, the supporting platform is fixed, and the indenter is loaded downward. The Snap-back process involves the restart operation, here, the restart function of ABAQUS is used to import the second stable state of the anti-symmetric cylindrical shell obtained during the snap-through process, and then align the indenter and load it downward. Furthermore, the anti-symmetric cylindrical shell involves large geometric nonlinear [33,34] deformation during the steady state transition, so it is necessary to consider those specific conditions in the numerical evaluations, activating the nonlinear large deformation options (e.g., setting ‘Nlgeom’ to ‘On’ in either ABAQUS or ANSYS software packages); at the same time, turning on the automatic stabilization function, that is, ‘specify dissipated energy fraction’ of ‘automatic stabilization’ option, which can make the finite element calculation process easier to converge [35]. Since the bistable shell problem belongs to the deformation analysis of the composite laminated thin shell structure, the quadrilateral shell element (S4R) with linear, finite film strain, and reduced integration is selected as the mesh element. “Hard contact” is used to simulate the normal behavior of bistable cylindrical shell. The material properties of the layer is shown in Table 1.

In addition, the mesh density of the anti-symmetric cylindrical shell simulation calculation has a great influence on the stable characteristics of the cylindrical shell. In the simulation, the anti-symmetric cylindrical shell controls the mesh density by the number of seeds. If the grid density is large (the number of grids is small), the calculation result is inaccurate; if the grid density is small (the number of grids is large), the calculation time is longer. As the geometric size of the cylindrical shell changes during the reanalysis of the model, the number of cylindrical shell meshes will also change accordingly. Therefore, the determination of the cylindrical shell mesh density can ensure the validity and speed of the calculation. Figure 1b,c show the grid independence analysis of anti-symmetric cylindrical shell in first and second steady state transition load, respectively. The anti-symmetric cylindrical shell with longitudinal length of 52.4 mm, single layer thickness of 0.10 mm, initial natural radius of 25 mm, central angle of 110°, and layer angle of 45° are selected in this paper. When the number of grids is greater than 5000, the two transition loads of the anti-symmetric cylindrical shell hardly change, so the number of grids of the cylindrical shell is selected as 5000, and the number of seeds is 35.

### 2.2. Problem Description

Bionic design is used to design artificially some wonderful structures or phenomena that naturally exist in nature, and which can play special effects on some special occasions. Among them, the anti-symmetric cylindrical shell bionic gripper structure inspired by the Venus flytrap’s grasping mechanism is such a special structure [36,37], which has fast response and high grasping power, and it can well imitate the grabbing action of flytraps. In order to better utilize the characteristics of the bionic gripper structure, the optimal design of the anti-symmetric cylindrical shell is indispensable. In this paper, the stable transition forces of the anti-symmetric cylindrical shell are taken as the optimization objectives, and the maximum strain after the stable transition is used as the constraint condition for optimization [38].

**Figure 2:**
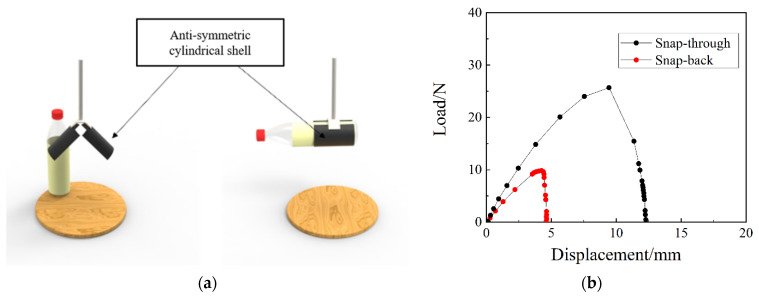
Anti-symmetric cylindrical shells: (**a**) prototype of the robotic gripper, (**b**) load-displacement curves.

Figure 2a shows the schematic diagram of the bionic gripper structure [39] based on the anti-symmetric cylindrical shell, which has two states of open to be grasped and closed, and it has faster response, higher clamping force, and no need for external continuity energy input to maintain structural stability. In order to make the bionic gripper structure easier to be driven, we choose the stable transition load during the snap-through process of the anti-symmetric cylindrical shell as an optimization goal, to minimize the stable transition load. At the same time, in order to make the bionic gripper structure with high load-bearing capacity and not being easily released after the grasping action is completed, we take the stable transition load during the snap-back process of the anti-symmetric cylindrical shell as another optimization goal, to maximize the stable transition load. The load-displacement curve of the anti-symmetric cylindrical shell is shown in the Figure 2b.

**Figure 3:**
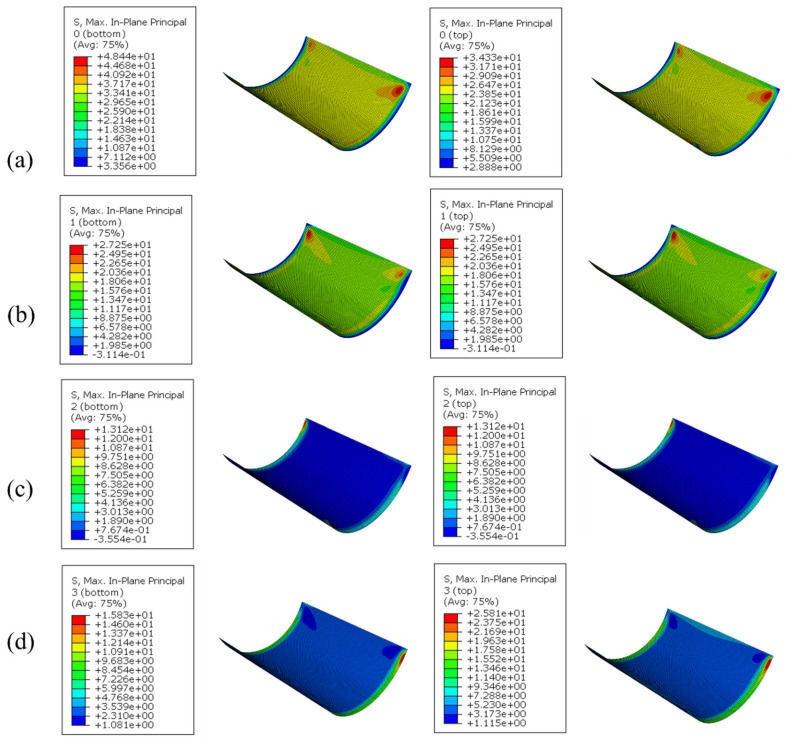
Distribution of maximum principal plane stress nephogram of each layer of anti-symmetric cylindrical shell in the second stable state: (**a**) the first layer, (**b**) the second layer, (**c**) the third layer, (**d**) the fourth layer.

When the anti-symmetric cylindrical shell is transformed to the second stable state, the shell will have some stress concentration phenomenon, which will have a great influence on the stable characteristics of the anti-symmetric cylindrical shell. Therefore, it is required that the maximum principal plane stress of the anti-symmetric cylindrical shell in the second stable state should not exceed a certain limit during the entire design process, which is a constraint imposed during the entire optimization design process. The stress cloud diagram of each layer of the anti-symmetric cylindrical shell is shown in Figure 3. It can be seen that the stress levels of the different layers of the anti-symmetric cylindrical shell are different, and the stress values of the upper and lower layers of the same layer are also different due to the shell element with a certain thickness itself. There is a global maximum principal plane stress value inside the outermost layer, so as long as the maximum principal plane stress at the bottom of the layer satisfies the constraint condition, then the stress of the entire cylindrical shell can be guaranteed to meet the condition.

### 2.3. Design Variable

**Figure 4:**
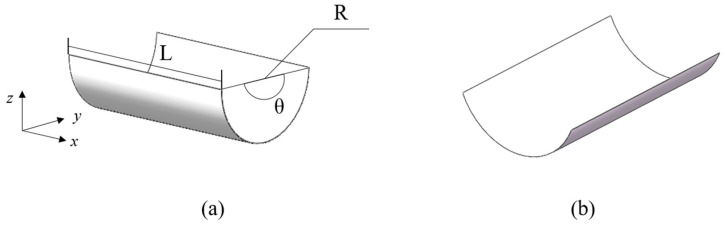
Two configurations of anti-symmetric shells: (**a**) initial steady state, (**b**) second steady state.

The bionic gripper structure is mainly composed of two identical anti-symmetric cylindrical shells, as shown in Figure 4. The anti-symmetric cylindrical shell has two stable states [40], deformed as a whole when one steady state changes to another steady state; the original straight side becomes an arc side and the original arc side becomes a straight side, yet the direction of curvature of the arc does not change in the two steady states. The main design variables [41] are longitudinal length *L*, layer thickness *t*, initial natural radius *R*, central angle *θ*, and layer angle *α*. Here, we choose the stacking sequence and layer number as [*α*/−*α*/*α*/−*α*].

There is a certain coupling relationship between the lateral length of the anti-symmetric cylindrical shell, the central angle and the initial natural radius, and the lateral length can be determined by these two design variables. Therefore, the layer thickness *t*, the initial natural radius *R*, the central angle *θ*, and the layer angle *α* are selected for the stable characteristic analysis, and the lateral length is not analyzed for the stable characteristic.

#### 2.3.1. The Layer Thickness *t*

**Figure 5:**
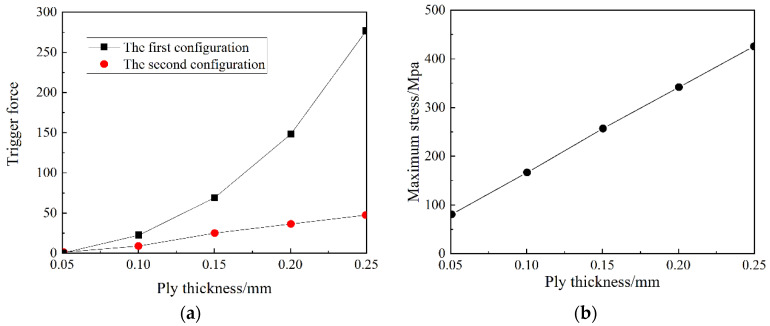
The influence of layer thickness on: (**a**) the transition load, and (**b**) maximum principal plane stress.

When analyzing the layer thickness of the anti-symmetric cylindrical shell, only the layer thickness is changed, while the other design variables of the shell remain unchanged. Figure 5 shows the influence of the layer thickness on the transition load and the maximum principal plane stress in the snap-through and snap-back processes of an anti-symmetric cylindrical shell. It can be seen from Figure 5a that the transition load gradually increases as the layer thickness increases. Among them, the first stable transition load value in the snap-through process changes greatly, while the second stable transition load value during the snap-back process changes slightly. It can be seen from Figure 5b that the maximum principal plane stress of the anti-symmetric cylindrical shell increases linearly as the thickness of the layer increases.

#### 2.3.2. The Initial Natural Radius *R*

**Figure 6:**
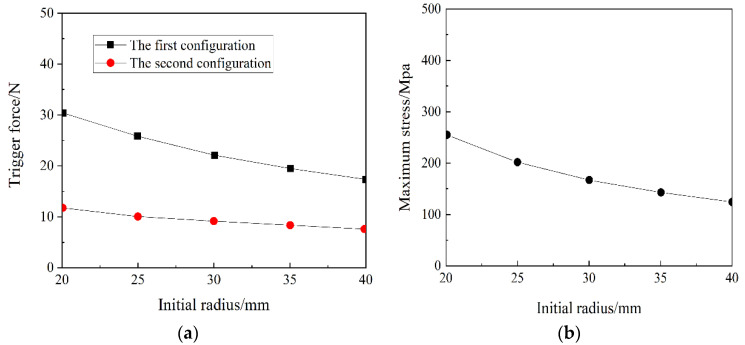
The influence of initial radius on: (**a**) the transition load, and (**b**) maximum principal plane stress.

The initial radius is one of the differences between the anti-symmetric cylindrical shell and the orthogonal laminated plate. Orthogonal laminates are laid on a flat plate and the structure flexes after curing, while the anti-symmetric cylindrical shell has a certain curvature at the beginning-the initial curvature. Figure 6 shows the effect of the initial radius on the transition load and the maximum principal plane stress in the snap-through and snap-back processes of an anti-symmetric cylindrical shell. It can be seen from Figure 6a that the two transition loads of the anti-symmetric cylindrical shell gradually decrease as the initial radius increases. The magnitude of the decrease in the first stable transition load of the anti-symmetric cylindrical shell is greater than that of the second stable transition load. It can be seen from Figure 6b that the maximum principal plane stress continues to decrease as the initial radius of the anti-symmetric cylindrical shell increases.

#### 2.3.3. The Central Angle *θ*

**Figure 7:**
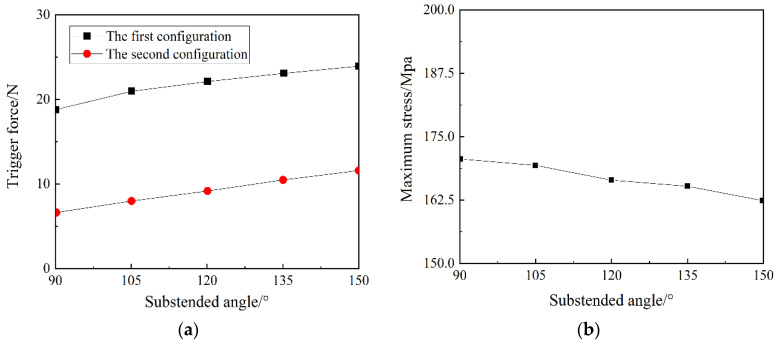
The influence of central angle: (**a**) the transition load, and (**b**) maximum principal plane stress.

The central angle determines the proportion of the space occupied by the anti-symmetric cylindrical shell in the first stable state. The larger the central angle, the larger the space occupied by the anti-symmetric cylindrical shell. Figure 7 shows the influence of the central angle on the transition load and the maximum principal plane stress in the snap-through and snap-back processes of an anti-symmetric cylindrical shell. It can be seen from Figure 7a that the transition load increases with the increase of the central angle, and the growth trends of the two stable transition loads are very close. It can be seen from Figure 7b that there is a negative correlation between the maximum principal plane stress and the central angle.

#### 2.3.4. The Layer Angle *α*

**Figure 8:**
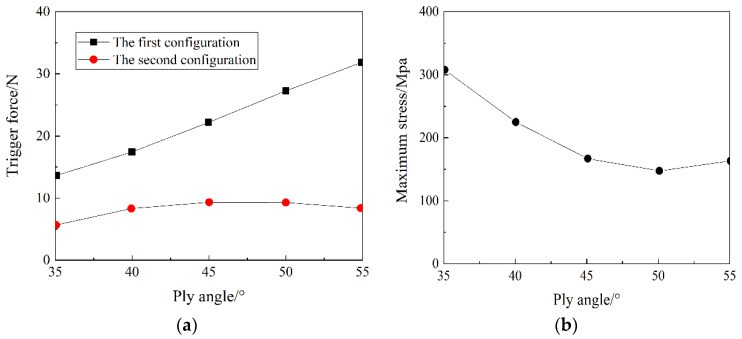
The influence of layer angle on: (**a**) the transition load, and (**b**) maximum principal plane stress.

Figure 8 shows the effect of the layer angle on the transition load and the maximum principal plane stress in the snap-through and snap-back processes of an anti-symmetric cylindrical shell. It can be seen from Figure 8a that with the increase of the layering angle, the first steady state transition load of the anti-symmetric cylindrical shell gradually increases, while the second steady state transition load of the anti-symmetric cylindrical shell increases first and then decrease. It can be seen from Figure 8b that with the increase of the ply angle, the maximum principal plane stress of the anti-symmetric cylindrical shell gradually decreases first and then rises.

#### 2.3.5. Analysis of the Influence Level of Design Variables

**Figure 9:**
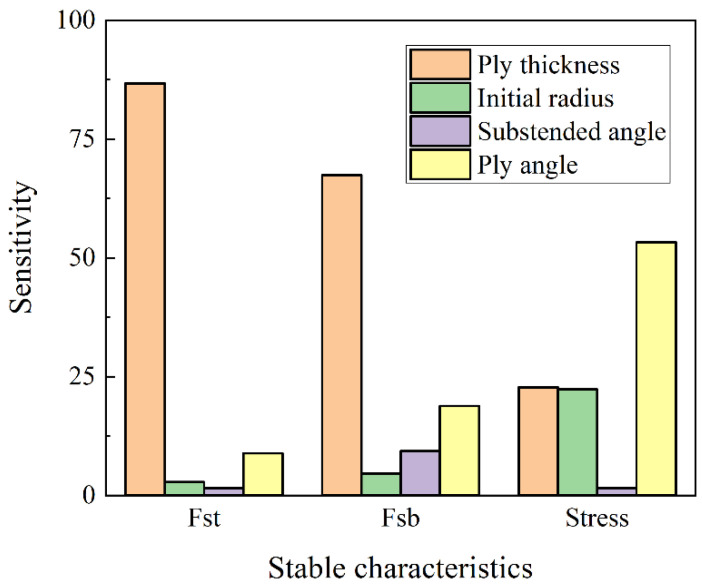
The level of influence of different design variables on the stable characteristics of anti-symmetric cylindrical shells.

From the above analysis, it can be seen that the four design variables of the anti-symmetric cylindrical shell (layer thickness *t*, initial natural radius *R*, central angle *θ*, and layer angle *α*) have significant influence on its two stable transition loads and maximum principal plane stress. Figure 9 shows the level of influence of four design variables on the stable characteristics of anti-symmetric cylindrical shells. Among them, the biggest influence on the first stable transition load is the layer thickness of 86.70%, and the smallest impact is the central angle of 1.56%. The biggest influence on the second stable transition load is the layer thickness of 67.35%, and the smallest influence is the initial radius of 4.52%. The biggest influence on the maximum principal plane stress is the ply angle of 53.24%, and the smallest influence is the central angle of 1.59%.

## 3. Multi-Objective Optimization Model Based on NSGA-Ⅱ Algorithm

### 3.1. Specimen Point Collection

**Figure 10:**
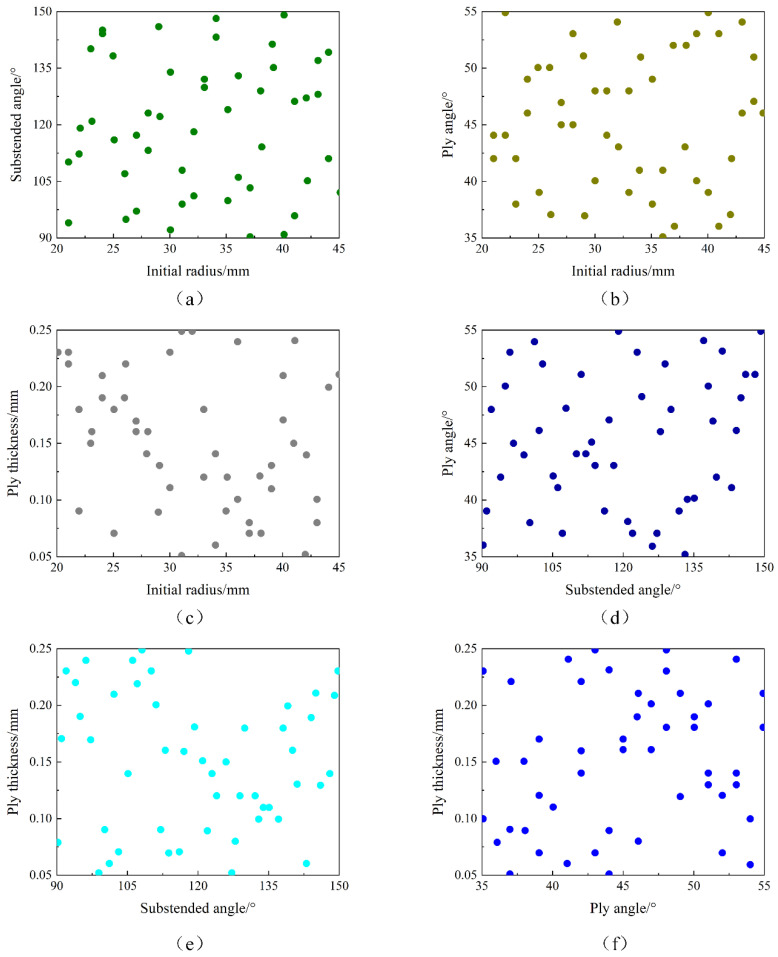
Specimen point distribution relationship of design variables for anti-symmetric cylindrical shells: (**a**) initial radius and angle of embrace, (**b**) initial radius and layer angle, (**c**) initial radius and layer thickness, (**d**) central angle and layer Angle, (**e**) central angle and layer thickness, (**f**) layer angle and layer thickness.

In order to obtain specimen points accurately and efficiently, the Latin hypercube experimental design method is selected to collect specimen points after determining the above four design variables. Generally, the more specimen points of the experimental design, the more accurate the approximate model based on the specimen points. However, due to the complex calculation model and calculation process of anti-symmetric cylindrical shells, and the time spent on a single sampling being longer, therefore, the number of sampling points must be obtained by weighing the accuracy of the model and the calculation cost in order to save time and calculation cost. In this paper, 50 specimen points are collected by the Latin Hypercube experimental design method to establish a second-order response surface model. Figure 10 shows the spatial distribution of specimen points collected by the Latin Hypercube experimental design method by the Isight software. The experimental design module in the Isight software can effectively explore the design space used to establish an approximate model, and the modeling and calculation speed are fast. On the whole, the design specimen group derived from the experimental design has a relatively random distribution in the design space, and there is no large blank part or agglomeration, so it can be better distributed in the design space and representative, and can play a good effect on the establishment of approximate models. RSM approximation based on a polynomial fit via the least square regression of the output parameters to the input parameters. Depending on the selected order of the polynomial (linear, quadratic, cubic, quartic), initialization of the approximation will require a certain number of design points to be evaluated. The component being approximated can be executed multiple times to collect the required data. Alternatively, a data file can serve as the initialization source. In this paper, 50 sample points are collected and simulated, the finite element results are used as the output of the response surface model, and the corresponding geometric parameters are used as the output of the response surface model. The error of the response surface model is reflected by the minimum complex relationship, the maximum root mean square, the maximum average value, and the maximum value.

### 3.2. Approximate Model

**Figure 11:**
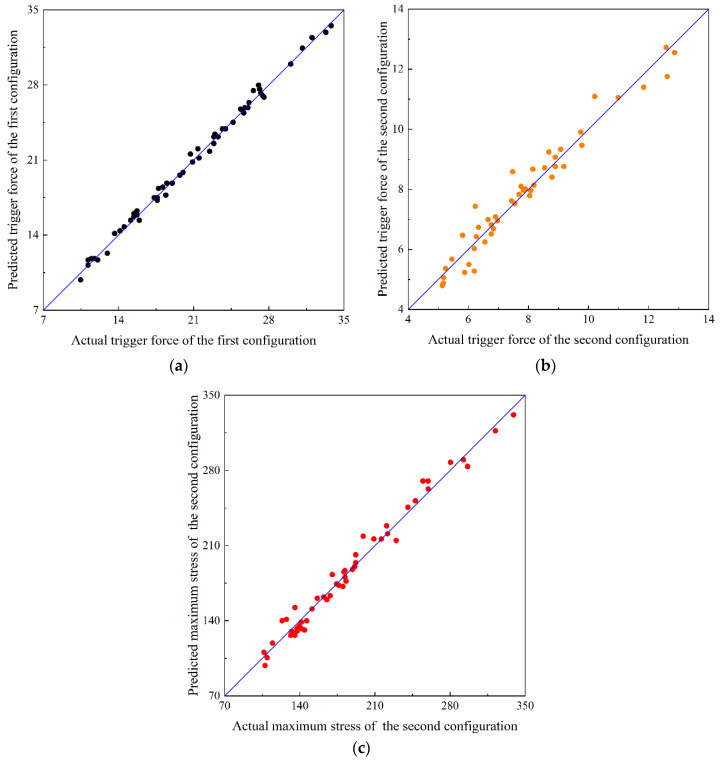
Comparison of the approximation model of three optimization indicators with actual values: (**a**) first stable transition load, (**b**) second stable transition load, (**c**) maximum principal plane stress.

Figure 11 shows the comparison between the predicted value and the actual value of the approximate model. The closer the midpoint of the coordinate system is to the 45° line, the closer the predicted value and the actual value. It can be seen from the figure that the predicted value of the second-order response surface approximation model for the three optimization indexes of the first steady state transition load, the second steady state transition load and the maximum principal plane stress are close to the actual values.

Table 2 shows the common indicators and standard values for the accuracy evaluation of the second-order response surface approximation model of the three optimization indicators. It can be seen from the table that the minimum complex relationship (*R*^2^) of the second-order response surface approximate model of the three optimization indicators is 0.9571, which is greater than the standard value of 0.9. The maximum root mean square (RMSE) is 0.0434, which is less than the standard value of 0.2. The maximum average value ($\bar{R}$) is 0.0329, which is less than the standard value of 0.2. The maximum value (*R_max_*) is 0.1313, which is less than the standard value of 0.3. Therefore, the second-order response surface approximation models of the above three optimization indexes all meet the requirements.

### 3.3. Optimization Results

The objective function [42,43] of this paper is to minimize the first steady-state transition load of the anti-symmetric cylindrical shell and maximize the second stable transition load. At the same time, in order to prevent excessive stress concentration in the anti-symmetric cylindrical shell, the maximum stress value of the ply when the anti-symmetric cylindrical shell is in the second stable state is taken as a constraint condition. Selecting the value range of the corresponding design variables (layup thickness *t*, initial natural radius *R*, central angle *θ* and layup angle *α*) according to the common anti-symmetric cylindrical shell size in the experiment, see Formula (1):

(1) $$Opt.\;\;\;\left\{ \begin{array}{l} \;\;\;\;Maximize:F_{\mathrm{sb}}\\ \;\;\;\;Minimize:F_{st}\\ \;\;\;\;Constraint\;condition:\;\;\;\mathrm{Stress}<\left|S\right|\\ \;\;\;\;\;\;\;\;\;\;\;\;\;\;\;\;\;\;\;\;\;\;Initial\;natural\;radius\;R\;\in [20,45]\\ \;\;\;\;\;\;\;\;\;\;\;\;\;\;\;\;\;\;\;\;\;\;Central\;angle\;\theta \;\in [90,150]\\ \;\;\;\;\;\;\;\;\;\;\;\;\;\;\;\;\;\;\;\;\;\;Layup\;angle\;\alpha \;\in [35,55]\\ \;\;\;\;\;\;\;\;\;\;\;\;\;\;\;\;\;\;\;\;\;\;Layup\;thickness\;t\;\in (0.06,0.08,0.10,0.12,0.15,0.18,0.2,0.25) \end{array}\right.$$

**Figure 12:**
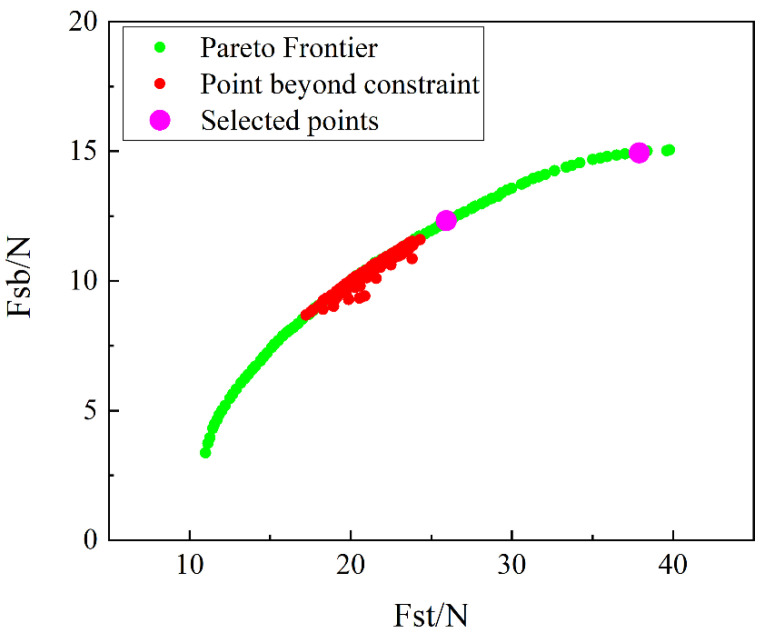
Pareto fronts of *F_st_* and *F_sb_* for anti-symmetric cylindrical shells.

The anti-symmetric cylindrical shell is optimized through the NSGA-II algorithm [44,45,46,47]. The total population is set to 100, the number of iterations is 50, the crossover rate is 0.9, and the maximum number of calculations of the model is 5000, and the result is shown in Figure 12. Among them, the boundary line formed by the green dots is the Pareto frontier line [48]. Two optimal solutions according to laboratory conditions and manufacturing process requirements are selected on the Pareto frontier line, which are represented in pink in the figure. At the same time, there are some red dots on the Pareto front line, which are design points that do not meet the constraints. To prepare the bistable cylindrical shell, the carbon fiber prepreg is cut to the required size and then laid on a cylindrical aluminum mold. After the laying is completed, a vacuum bag is placed on the outer layer of the mold to ensure that the carbon fiber does not come into contact with the air during high temperature and high pressure curing in the autoclave. After three hours of high temperature and high pressure curing, the cylindrical mold was left to cool at room temperature for half an hour and then the carbon fiber bistable shell was removed from the mold as an experimental specimen. An Instron tensile testing machine is used to test the mechanical properties of the experimental samples. In this paper, three samples are prepared, one initial design is used as the control group, and two optimized results are selected as the optimal design to prove the accuracy and effectiveness of this optimization.

Table 3 shows the specific parameters of the design variables of the initial specimen and the optimized specimen point of the anti-symmetric cylindrical shell. The selection of the initial specimen points is random, the layer angle is (45°/−45°/45°/−45°), the layer thickness is 0.10 mm, the central angle is 120°, and the initial radius is 25 mm.

Table 4 shows the comparison of the finite element solution and response surface solution of the initial specimen and the optimized specimen point of the anti-symmetric cylindrical shell. It can be found that the response surface approximation model solution of the specimen points is relatively close to the finite element solution. Among them, the largest relative error value appears in the second stable transition load of specimen 2, and the maximum relative error is 9.0%. Therefore, the approximate model prediction result is reasonable.

By comparing the initial specimen points, the pros and cons of the optimized results of the selected specimen points can be obtained. Table 5 shows the stable transition load of two specimens after optimization for finite element results based on response surface method. A positive sign indicates that the optimization result is increased relative to the load of the initial specimen, and a negative sign indicates that the optimization result is reduced. It can be seen from the table that compared with the initial specimen, the first stable load of specimen 1 increases, which violates the optimization goal, and the second stable load increases, which is consistent with the optimization goal. Compared with the initial specimen, the first stable load of specimen 2 decreases and the second stable load increases, which are consistent with the optimization goal.

## 4. Experimental Verification

**Figure 13:**
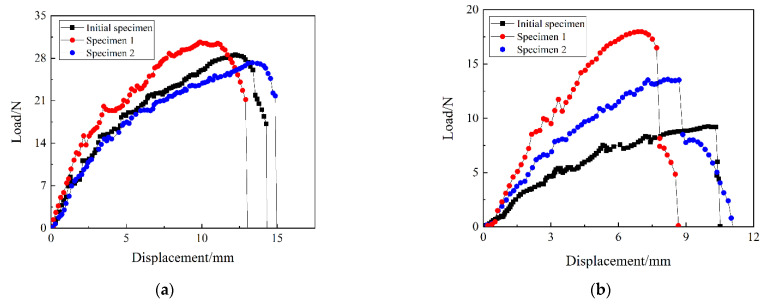

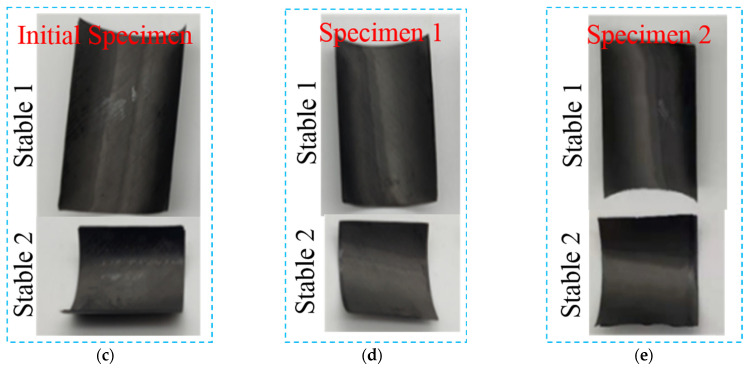
Load-displacement curves and specimens prepared in the experiment: (**a**) the snap-through process, (**b**) the snap-back process, (**c**) initial specimen, (**d**) specimen 1, (**e**) specimen 2.

Figure 13 shows the load-displacement curves of three types of anti-symmetric cylindrical shells measured by a universal tensile testing machine and prepared specimen in the experiment. The direction of curvature of the two stable configurations of the anti-symmetric cylindrical shell does not change with the stable transition. In order to compare the experimental and finite element results more clearly, Table 6 lists the experimental and finite element results of the two stable transition loads of the three specimens. It can be seen from the table that the maximum relative error between the finite element results and the experimental results is 17.22%. Here, the error is mainly due to the torsion phenomenon that is prone to occur during the preparation of the antisymmetric sample. The specific reasons for the error may be: (1) The laying of the two prepreg cloth layers is not completely aligned when preparing the specimen; (2) The prepreg cloth laying is difficult to be completely centered in the mold, causing the anti-symmetric cylindrical shell to twist; (3) The anti-symmetrical cylindrical shell may slip when in contact with the indenter, during the test on the tensile testing machine.

Table 7 shows the stable transition load of two specimens after optimization for experimental results based on response surface method. It can be seen from the table that the optimization trend of the experimental results of specimen 1 and specimen 2 is consistent with the optimization trend of finite element results.

## 5. Conclusions

In this paper, a finite element method based on two-point loading method and approximate model are proposed to optimize the design parameters of the anti-symmetric cylindrical shell for bionic gripper structure application, and the experiment verification method verifies the accuracy of the optimization results. The conclusions are as follows:

(1) The geometric parameters of the anti-symmetric cylindrical shell have a great influence on its configuration and driving force.

(2) Good agreement is obtained between finite element analysis and experimental results. The error between the finite element and the experiment is controlled within 20%.

(3) The approximate model established in this paper has high accuracy to predict the mechanical response of anti-symmetric shells with different geometric parameters. The high precision of the response surface model is reflected by four standard values and the values of the indicators are all within the acceptable range.

(4) The finite element simulation and experiment of the optimization results verify the accuracy and feasibility of the multi-objective optimization method in this paper. The experimental results show that transition load in snap-back can be increased by a maximum of 93.6%.

## Figures and Tables

**Table 1:** The material properties of the anti-symmetric cylindrical shell [31].

Properties	Values	Units
Longitudinal Young’s modulus (*E*_11_)	130	GPa
Transverse Young’s modulus (*E*_22_)	10	GPa
Poisson’s ratio (*μ*)	0.3	
Shear modulus (*G*_12_)	4.4	GPa
Longitudinal thermal expansion coefficient (*α*_11_)	−0.018	10–6/°C
Transverse thermal expansion coefficient (*α*_22_)	30	10–6/°C

**Table 2:** Evaluation of the response surface approximation model of three optimization indicators.

Indicators (Standard Values)	*F_st_*	*F_sb_*	*S*_max_
*R*^2^ (>0.9)	0.9962	0.9571	0.9760
*RMSE* (<0.2)	0.0130	0.0434	0.0297
$\bar{R}$ (<0.2)	0.0101	0.0329	0.0215
$R_{max}$ (<0.3)	0.0519	0.1247	0.1313

**Table 3:** The specific parameters of design variables of initial specimen and the optimized specimen point.

Parameters	*θ* (°)	*R* (mm)	*γ* (°)	*t* (mm)
Initial specimen	45	25	110	0.10
Specimen 1	45.7	20	149.9	0.10
Specimen 2	40.8	25	120	0.10

**Table 4:** Relative error between the FEA and RSM results for specimen point of anti-symmetric shells.

Parameters	Finite Element Solution			Response Surface Solution			Relative Error		
	F_st_ (N)	F_sb_ (N)	S (MPa)	F_st_ (N)	F_sb_ (N)	S (MPa)	F_st_ (%)	F_sb_ (%)	S (%)
Initial specimen	27.0	10.5	212.1	25.8	9.8	201.8	4.8	7.1	5.1
Specimen 1	33.8	14.9	235.9	34.9	14.9	241.5	3.2	0.5	2.3
Specimen 2	23.3	12.3	246.6	22.9	11.2	250.6	2.1	9.0	1.6

**Table 5:** Stable transition load of two specimens after optimization for finite element results based on response surface method.

Parameters	F_st_	F_sb_
Specimen 1	25.14%	42.34%
Specimen 2	−13.52%	16.72%

**Table 6:** Comparison between the EXP and FE results of two transition loads of three specimens.

		Exp (N)	FE (N)	Relative Error (%)
Transition load in Snap-through	Initial specimen	28.6	27.0	5.59
	Specimen 1	30.0	34.9	12.67
	Specimen 2	27.0	22.9	13.71
Transition load in Snap-back	Initial specimen	9.3	10.5	12.90
	Specimen 1	18	14.9	17.22
	Specimen 2	13.5	11.2	8.89

**Table 7:** Stable transition load of two specimens after optimization for experimental results based on response surface method.

	F_st_	F_sb_
Specimen 1	4.90%	93.60%
Specimen 2	−5.59%	45.20%

## Data Availability

Excluding this statement.
